# The association between dietary quality index- international and metabolic risk factors in RaNCD cohort study

**DOI:** 10.1186/s12889-024-20513-w

**Published:** 2024-10-30

**Authors:** Zahra Mokhtari, Hadi Abdollahzad, Neda Izadi, Shahab Rezaeian, Farid Najafi, Yahya Pasdar

**Affiliations:** 1 Student Research Committee, Kermanshah University of Medical Sciences, Kermanshah, Iran; 2 Department of Nutrition, School of Medicine, Urmia University of Medical Sciences, Urmia, Iran; 3 Research Center for Social Determinants of Health, Research Institute for Endocrine Sciences, Shahid Beheshti University of Medical Sciences, Tehran, Iran; 4 Infectious Diseases Research Center, Health Institute, Kermanshah University of Medical Sciences, Kermanshah, Iran; 5 Research Center for Environmental Determinants of Health (RCEDH), Health Institute, Kermanshah University of Medical Sciences, Kermanshah, Iran; 6 Nutritional Sciences Department, School of Nutritional Sciences and Food Technology, Isar Square, Kermanshah Iran

**Keywords:** Non-communicable disease, Metabolic risk factors, Dietary quality index- international (DQI-I), Diet, Persian cohort

## Abstract

**Background:**

Non-communicable diseases (NCDs) are the leading cause of death around the world. The Dietary Quality Index-International (DQI-I) is one of the indicators that shows changes in diet and its association with NCDs. The aim of this study is to measure the association between the DQI-I and major metabolic risk factors.

**Methods:**

This study is a cross-sectional study based on data collected in the first phase of the prospective cohort study on Ravansar non-communicable diseases (RaNCD). To perform clinical and biochemical tests such as lipid profile, blood glucose and liver enzymes, blood samples were collected using standard vacutainer blood collection techniques. Information from the food frequency questionnaire containing 118 food items was used to score each person’s DQI-I. Binary logistic regression was used to determine the association between the DQI-I tertile and the metabolic risk factors. Linear regression was also used for the association between subgroups of DQI-I score and the metabolic risk factors.

**Results:**

The total number of subjects in this study was 7,115, with a mean age of 47.14 ± 8.29 years. Dietary quality was generally poor, with 37.03% in the lowest tertile. Men had better dietary quality than women. Anthropometric measures, blood pressure, triglycerides and blood glucose were lower in the lowest tertile of dietary quality. A one-unit increase in the total DQI-I resulted in a 0.19 decrease in total cholesterol. Higher dietary quality was associated with a 22% increased risk of high triglycerides, a 19% increased risk of low high-density density lipoprotein, a 5% decreased risk of elevated low-density lipoprotein, a 42% increased risk of high blood pressure, and a 99% increased risk of high fasting blood glucose. Higher dietary quality was also associated with a 33% increased risk of overweight/obesity.

**Conclusion:**

The study found that participants had poor dietary quality, with some favorable metabolic outcomes in the lowest tertile, but concerning associations in the highest tertile, including increased risk for high triglycerides, blood pressure, and obesity. The complex associations suggest that balanced, multifaceted interventions are needed.

## Introduction

Non-communicable diseases (NCDs) are the leading cause of death worldwide [1]. NCDs lead to the death of 41 million people each year, representing 74% of all deaths in the world [1]. Most deaths related to NCDs are attributed to major risk factors, which can generally be divided into two categories: metabolic factors and behavioral factors [2]. Metabolic risk factors, including hypertension, high fasting glucose, dyslipidemia, and obesity or overweight, are critical indicators of an individual’s likelihood of developing chronic diseases such as type 2 diabetes (T2D) and cardiovascular disorders [2, 3]. These factors are increasingly prevalent in modern society, driven by lifestyle changes, dietary habits, and sedentary behaviors.

High blood pressure is one of the primary mtabolic risk factors worldwide [4]. Approximately 15–20% of individuals with hypertension will develop diabetes, 30% will experience dyslipidemia, and overall, 50% are likely to develop cardiovascular disease in the future [4]. The global prevalence of diabetes is estimated to be 9.3% (463 million people) in 2019, reaching 10.2% in 2030 and 10.9% in 2045 [5]. The prevalence of obesity has increased dramatically over the last 50 years. Studies show that overweight people have a 40% higher risk of death and obese people have a 300% higher risk of death [6].

Diet can dramatically change the health status of the general population [7]. Proper nutrition is known to be an epigenetic factor that can be applied in short periods of time with lasting effects [8]. Various factors lead to inflammation, but diet and obesity are the most important triggers of inflammation [9]. Dietary quality is thought to have an impact on the development of NCDs [10].

The Dietary Quality Index-International (DQI-I) is one of the types of indices created to show changes in diet and its association with metabolic factors [11]. The DQI-I was developed to be used for international comparisons because it focuses not only on NCDs but also on malnutrition problems [11]. Studies have shown that a higher DQI-I score is associated with a lower risk of metabolic syndrome, obesity, T2D, and cardiovascular disease [12–14]. In addition, a cross-sectional study comparing DQI-I ability and identifying risk factors for NCDs showed that DQI-I score was inversely related to total cholesterol (TC), low-density lipoprotein cholesterol (LDL-C), high-density lipoprotein cholesterol (HDL-C), central obesity, and waist circumference (WC), but there was no association between blood glucose parameters and DQI-I score [15].

This suggests that adherence to a high-quality diet, as measured by the DQI-I, may have a protective effect against the development of metabolic risk factors. Furthermore, the DQI-I provides valuable insights into the relationship between dietary patterns and metabolic health and thus potential strategies for the prevention and management of metabolic disorders. Therefore, in this study, we sought to investigate the association between dietary quality and risk factors for NCDs.

## Method

### Study population

The present study is a cross-sectional study conducted on the basis of data collected in the first phase of the prospective cohort study of Ravansar non-communicable diseases (RaNCD) [16]. The RaNCD cohort is considered a branch of the Prospective Epidemiological Research Studies in IrAN (PERSIAN) and is designed to determine the incidence of NCDs [17]. In the this study, all participants of the Ravansar cohort, 10,047 individuals aged 35 to 65 years, were included in the study. In addition, individuals who reported abnormal daily energy intake (as defined less than 800 and more than 4,200 kcal/day), those with missing data regarding the main exposure and outcomes, and individuals with chronic diseases—including cancer, renal failure, chronic lung disease, rheumatic disease, and gallstones—were excluded from the study (*n* = 2,932).

### Measurements

All information about the participants was collected using a digital questionnaire administered by trained experts at the cohort center. Individuals’ demographic data, including age, gender, marital status and education level, were collected using standard Persian cohort questionnaires. Socioeconomic status (SES) includes employment status, education level, employment history, place of residence, number of domestic and international trips, access to landline and cell phones, and internet and cell phone use.

The PERSIAN Cohort standard physical activity questionnaire was used to assess participants’ physical activity levels. The questionnaire contained 22 questions about the person’s daily activities, and finally the questionnaire data were extracted and used based on the metabolic equivalent of work per hour per day (MET/h/day).

Body measurements were obtained using the InBody 770 body composition analysis device. Weight measurements were recorded using the same device, ensuring a precision of 100 g. Height was measured using the Biospace Co. Stadiometer 370 automated device, and the recorded heights were then documented in centimeters in the InBody 770 device. Body mass index (BMI) was calculated using the formula weight (kg)/height^2^ (m).

The blood pressure measurements followed the standard methodology of the Persian cohort. To perform clinical and biochemical tests such as lipid profile, blood glucose and liver enzymes, blood samples were collected using standard vacutainer blood collection techniques, with participants assuming a seated position and a tourniquet applied. Further details have already been published in the RaNCD cohort study protocol [16].

### Metabolic risk factors

The high-value metabolic risk factors were defined as follows: A BMI greater than 25 kg/m^2^, a WC greater than 95 cm, a waist-to-hip ratio (WHR) greater than 0.95, a waist-to-height ratio (WHtR) greater than 0.5, and a body fat percentage (BFP) greater than 29.5%. High-risk lipid levels include TC above  200 mg/dl, triglycerides (TG) above  150 mg/dl, HDL-C above 45 mg/dl and LDL-C above  100 mg/dl. Elevated blood pressure is defined as systolic blood pressure (SBP) of more than 12 mmHg and diastolic blood pressure (DBP) of more than 8 mmHg. Fasting blood sugar (FBS) levels above 125 mg/dl are considered high risk. In addition, high levels of liver enzymes include aspartate aminotransferase (SGOT) below 9.5 or above 39.5 IU/L, alanine aminotransferase (SGPT) below 7 or above 55 IU/L, alkaline phosphatase (ALP) above 350 IU/L and gamma-glutamyl transferase (GGT) above 63.5 IU/L.

### Nutritional assessment

Nutritional status was assessed using the Iranian National Food Frequency Questionnaire (FFQ) [18]. The FFQ asks about the consumption of food and beverages during the past year. This questionnaire contains 118 foods suitable for the Iranian population, including bread, grains, cereals, meat and meat products, milk and dairy products, vegetables, fruits, various oils and oilseeds, sugar, various foods, spices, and dietary supplements. Local foods were also included in the questionnaire. The amount of each food consumed was converted into grams per day (g/day) using Nutrition IV software to measure the daily intake of food. 

### Dietary quality index-international (DQI-I)

Information from the FFQ questionnaire containing 118 food items for each person was used to score each person’s DQI-I [18]. The overall structure and scoring system of the DQI-I were considered based on the study by Kim et al. [11]. The subgroups of this index consist of four categories, including the variety, adequacy, moderation and overall balance. This index was evaluated in two categories: first, overall variety, and second, variety of protein sources. Consumption of at least one meal per day from each of the five food groups (meat, poultry, fish/eggs, dairy, beans, grains, fruits and vegetables) determined the maximum total score for diversity. If none of these food groups were consumed, 3 out of 15 full points were deducted for each food group. To calculate the variety among protein sources, the highest score, 5 points, was awarded if the intake came from 3 different protein sources per day.

As the number of different sources decreased to 2, 1 and 0, the score also decreased to 3, 1 and 0 points respectively. In the adequacy subgroup, points were awarded for eight components in this category based on the percentage of achievement of the recommended intake on a continuous scale ranging from 0 points for no consumption (0%) to 5 points for 100% consumption, which was variable. The consumption of fruit, vegetables, cereals and fiber depended on the energy intake. Protein consumption was considered sufficient if it accounted for at least 10% of the total energy intake. The intakes for the highest adequacy level of iron, calcium and vitamin C were derived from the Dietary Reference Intakes (DRI), which differed by age and gender. In the “moderation” subgroup, nutrient intakes (total fat, saturated fat, cholesterol and sodium) were categorized into three levels depending on the impact on health. The lowest intake level received the highest score, 6 points; the highest level obtained was given the lowest score, 0 points, and the average level was given 3 points. A unique component of the DQI was the assessment of the consumption of foods with low nutrient density, so-called “empty-calorie foods” If the energy intake from empty-calorie foods was more than 10% of the total energy intake per day, the lowest score was awarded, and if it was less than 3%, the highest score was awarded. These foods consist mainly of carbohydrates and fats and have a lower content of nutrients such as vitamins, minerals and amino acids, such as ice cream and cookies, which fall into this category (80). The final category was overall balance, where the diet was analysed in terms of the proportion of energy sources and fatty acid composition. The ratio of energy sources and the composition of fatty acids were given scores of 6 and 4 respectively. The scores of each subgroup in each of the four main categories were summed, and the scores of all four categories were summed, resulting in a total DQI score ranging from 0 to 100 (0 being the lowest and 100 being the highest possible score).

Based on the completed questionnaires to investigate the risk factors and their association with the DQI-I, the participants were divided into three groups: Participants with poor diet (DQI-I score < 33.33), participants with average diet (33.33 < DQI-I score < 66.66) and participants with good diet (DQI-I score > 66.66).

### Statistical analysis

The statistical analyses were performed using STATA version 17 software. In the descriptive analysis, the quantitative variables were described as mean ± standard deviation (SD) and the qualitative variables as frequency (%). The t-test was used to compare the quantitative variables and the chi-square test was used to compare the qualitative variables in the groups studied, including metabolic risk factors. The chi-square test was used to assess the frequency of the categorical variables and the DQI-I tertiles of the participants. One-way analysis of variance (ANOVA) was performed to assess the differences between the continuous variables in the DQI-I tertiles. In addition, binary logistic regression was used in crude and adjusted models (age, sex, BMI and energy in model 1 and age, sex, BMI, energy, physical activity and socioeconomic status in model 2) to determine the strength of the association between the DQI-I tertile and the metabolic risk factors. Crude and adjusted odds ratios (OR) with 95% confidence intervals (CI) were reported. Linear regression was also used for the association between subgroups of DQI-I score and the metabolic risk factors. For this analysis, regression coefficient (β) and standard error of coefficient (S.E) were reported.

## Results

### Characteristics of the participants by DQI-I tertile

The total number of subjects in this study was 7,115, with a mean age of 47.14 ± 8.29 years. 44.41% were men and 55.59% were women. Most participants (37.03%) were in the first tertile, indicating poor dietary quality among participants. Men had better dietary quality than women, and the number of men increased from the first to the third tertile (*P* < 0.001). The quality of the women’s diet was very low, and about 42% of the women were in the first tertile, and the number of women in the second tertile was higher than in the third tertile. Individuals in the third tertile were younger. Most people in the third tertile lived in the city (34.37%). Smokers and people who consumed alcohol were mostly in the first tertile and were not statistically significant (P(smoke) = 0.528 and P(alcohol) = 0.388) (Table 1).

**Table 1:** Characteristics of the participants by DQI-I tertile

Variables		All	DQI-I tertile			*P*-value^*^
			Tertile 1 Cut point < 33.33	Tertile 2 66.66 > Cut point < 33.33	Tertile 3 Cut point > 66.66	
Number	-	7115	2635 (37.03)	2327 (32.71)	2153 (30.26)	-
Age	year	47.14	47.54 (8.39)	46.97 (8.29)	46.83 (8.19)	**0.007**
Sex	man	3160	977 (30.92)	1067 (33.77)	1116 (35.32)	**< 0.001**
	woman	3955	1658 (41.92)	1260 (31.68)	1037 (26.22)	
Marital status	single	335	176 (52.54)	98 (29.25)	61 (18.21)	**< 0.001**
	married	6362	2266 (35.62)	2099 (32.99)	1997 (31.39)	
	widow	327	152 (46.48)	102 (31.19)	73 (22.32)	
Residence	city	4114	1197 (29.10)	13.81 (31.57)	1536 (34.37)	**< 0.001**
	rural	3001	1436 (47.92)	946 (31.52)	617 (20.56)	
Socioeconomic status	poor	1420	570 (40.14)	490 (34.51)	360 (25.35)	**< 0.001**
	middle	1408	495 (35.16)	472 (33.52)	441 (31.32)	
	rich	1375	416 (30.25)	471 (34.25)	448 (35.49)	
Smoking status	no	2949	1051 (35.64)	955 (32.38)	943 (31.98)	0.528
	light	451	163 (36.14)	151 (33.48)	137 (30.38)	
	moderate	254	91 (35.83)	91 (83.35)	72 (28.35)	
	heavy	618	691 (32.72)	208 (33.666)	174 (28.16)	
Alcohol consumption	no	6810	2533 (37.20)	2224 (32.66)	2053 (30.15)	0.388
	yes	305	102 (33.44)	103 (33.77)	100 (32.79)	
Physical activity	light	2112	236 (38.19)	704 (33.33)	717 (33.95)	**< 0.001**
	middle	3481	1348 (38.72)	1074 (30.85)	1059 (30.42)	
	sever	1522	596 (39.16)	549 (36.07)	377 (24.77)	

Variables are reported as frequency and percentage. Age is reported as mean and standard deviation. ^*^Based on One-way ANOVA (for age) and Chi-square (for categorical variables) tests

### Anthropometric, clinical and biochemical characteristics by DQI-I tertile

The mean BMI was 27.41 ± 4.66 kg/m^2^, the WC was 97.06 ± 10.59 cm, the SBP was 107.65 ± 16.76 mmHg, and the DBP was 69.44 ± 9.79 mmHg. The mean of anthropometric indicators (BMI and WC), blood pressure (mmHg), TG (mg/dl), FBS (mg/dl) and gamma-glutamyl transferase enzyme (IU/L) were lower in the first tertile than in the third tertile, and all differences were statistically significant (*P* < 0.05). Total cholesterol (mg/dl) and LDL-C (mg/dl) were lower in the third tertile than in the first tertile, and TC was significant (P(TC) = 0.005) and P(LDL) = 0.069) (Table 2).

**Table 2:** The distribution of anthropometric, clinical and biochemical characteristics of the participants by DQI-I tertile

Variables	All	DQI-I tertile			*P*-value^*^
		Tertile 1 Cut point < 33.33	Tertile 2 33.33 < Cut point < 66.66	Tertile 3 Cut point > 66.66	
**Anthropometric characteristics**					
Body mass index (kg/m^2^)	27.41 ± 4.66	27.08 ± 4.80	27.47 ± 4.63	27.75 ± 4.47	**< 0.001**
Waist circumference (cm)	97.06 ± 10.59	96.32 ± 10.97	97.08 ± 10.61	97.92 ± 10.03	**< 0.001**
Body fat percentage (%)	34.04 ± 9.46	34.44 ± 9.50	33.85 ± 9.56	33.77 ± 9.29	**0.027**
Body fat mass (kg)	25.02 ± 9.55	24.63 ± 9.66	25.08 ± 9.61	25.45 ± 9.33	**0.012**
Skeletal muscle mass (kg)	26.09 ± 5.60	24.99 ± 5.39	26.42 ± 5.67	27.06 ± 5.57	**< 0.001**
Fat free mass (kg)	47.22 ± 9.26	45.41 ± 8.91	47.77 ± 9.35	48.84 ± 9.21	**< 0.001**
Skeletal lean mass (kg)	44.65 ± 8.77	42.95 ± 8.45	45.17 ± 8.86	86.18 ± 8.72	**< 0.001**
**Clinical characteristics**					
Systolic blood pressure (mmHg)	107.65 ± 16.76	106.58 ± 16.66	107.69 ± 16.67	108.93 ± 16.90	**< 0.001**
Diastolic blood pressure (mmHg)	69.44 ± 9.79	69.06 ± 9.66	69.34 ± 9.66	70.00 ± 10.06	**0.003**
Total cholesterol (mg/dl)	185.49 ± 38.09	187.41 ± 39.07	184.55 ± 37.20	184.1737.76	**0.005**
Triglyceride (mg/dl)	136.54 ± 81.83	131.92 ± 80.46	136.52 ± 80.81	142.22 ± 84.23	**< 0.001**
HDL cholesterol (mg/dl)	46.62 ± 11.30	48.12 ± 11.86	46.00 ± 10.84	45.64 ± 10.87	**< 0.001**
LDL cholesterol (mg/dl)	102.06 ± 11.30	102.96 ± 25.86	101.62 ± 24.89	101.43 ± 25.51	0.069
Fast blood sugar (mg/dl)	97.18 ± 30.19	95.83 ± 27.35	95.31 ± 27.03	100.83 ± 35.89	**< 0.001**
**Biochemical characteristics**					
Aspartate aminotransferase (IU/L)	21.31 ± 9.15	21.26 ± 8.40	21.35 ± 10.87	21.34 ± 8.05	0.942
Alanine aminotransferase (IU/L)	24.45 ± 14.40	23.06 ± 13.31	24.52 ± 14.85	26.08 ± 15.01	**< 0.001**
Alkaline phosphatase (IU/L)	196.45 ± 60.83	198.14 ± 67.44	193.46 ± 55.82	197.60 ± 57.29	**0.015**
Gamma glutamyl transferase (IU/L)	23.96 ± 18.17	23.16 ± 17.27	23.32 ± 17.11	25.64 ± 20.15	**< 0.001**

Quantitative variables are reported with mean and standard deviation. ^*^Based on One-way ANOVA

### Main components and subgroups of DQI-I in different DQI-I tertile

Table 3 shows the important components and subgroups of DQI-I in the different DQI-I tertiles. For energy, carbohydrate, protein, fiber, calcium, vitamin C, and iron intake, an increasing trend was observed from the first to the third tertile, and then DQI-I subgroups such as variety and adequacy also increased. Saturated fat intake decreased from the first tertile to the third tertile (32.36 ± 17.71 g vs. 26.89 ± 12.84 g). The monounsaturated fatty acids (MUFAs) also decreased like the saturated fats, but the consumption of polyunsaturated fatty acids (PUFAs) was higher in the third tertile than in the first tertile (9.25 ± 4.10 g vs. 8.46 ± 4.55 g). In the DQI-I subgroups, variety, adequacy, moderation and overall balance were higher in the third tertile than in the first tertile, and they were all statistically significant (*P* < 0.001).

**Table 3:** The distribution of main components and subgroups of DQI-I in different DQI-I tertile

Variables	All	Tertile 1 Cut point < 33.33	Tertile 2 33.33 < Cut point < 66.66	Tertile 3 Cut point > 66.66	*P*-value
Energy	2495.14 ± 736.34	2276.9 ± 725.61	2581.49 ± 712.30	2668.90 ± 709.20	**< 0.001**
Carbohydrate	61.31 ± 6.19	58.74 ± 6.84	62.06 ± 5.73	63.63 ± 4.46	**< 0.001**
Protein	13.75 ± 2.17	13.14 ± 2.30	13.83 ± 2.08	14.42 ± 1.88	**< 0.001**
Fat	26.89 ± 5.94	29.75 ± 6.74	26.12 ± 5.15	24.23 ± 3.84	**< 0.001**
Saturated fat	29.93 ± 15.75	32.36 ± 17.71	29.99 ± 15.36	26.89 ± 12.84	**< 0.001**
MUFA	16.24 ± 8.43	18.62 ± 10.07	15.89 ± 7.56	13.71 ± 0.99	**< 0.001**
PUFA	8.90 ± 4.45	8.46 ± 4.55	9.07 ± 4.60	9.25 ± 4.10	**< 0.001**
Fiber	47.22 ± 20.53	36.97 ± 16.48	49.47 ± 18.93	57.35 ± 20.92	**< 0.001**
Iron	23.96 ± 9.61	20.10 ± 8.24	24.98 ± 9.14	27.60 ± 9.96	**< 0.001**
Calcium	733.52 ± 389.3	637.17 ± 392.3	757.82 ± 389.5	733.52 ± 358.2	**< 0.001**
Vitamin C	253.46 ± 138.04	183.93 ± 108.9	268.86 ± 130.7	321.89 ± 138.2	**< 0.001**
Sodium	2.32 ± 2.29	1.58 ± 2.06	2.21 ± 2.21	3.34 ± 2.27	**< 0.001**
Variety	12.67 ± 4.77	9.88 ± 4.67	13.12 ± 4.12	15.60 ± 3.44	**< 0.001**
Adequacy	33.28 ± 3.69	30.85 ± 4.19	34.07 ± 2.51	35.41 ± 2.05	**< 0.001**
Moderation	11.91 ± 5.53	9.09 ± 5.22	11.85 ± 4.64	15.44 ± 4.71	**< 0.001**
Overall balence	2.86 ± 2.20	2.05 ± 2.11	2.93 ± 2.17	3.73 ± 2.00	**< 0.001**

The scores of the components of the DQI-I are shown as mean and standard deviation. MUFA = Monounsaturated fatty acids, PUFA = Polyunsaturated fatty acids. The p-values are obtained through One-way ANOVA

### Association between metabolic risk factors and DQI-I tertile

Based on the results in Table 4 and after adjustment, the odds of overweight/obesity increases 1.33-fold when the DQI-I tertile is increased (OR = 1.33; 95% CI: 1.15–1.51). Other anthropometric indices, such as WC, WHR and BFP, showed a significant decrease in the models. In the univariable model, the odds of a high TG value was significantly 22 higher in the third tertile of the DQI-I than in the first tertile (OR = 1.22; 95% CI: 1.04–1.43). The odds of low HDL-C was significantly 19% higher in the third tertile than in the first tertile (OR = 1.19; 95% CI: 1.05–1.33). After adjustment, the odds of high blood pressure (systolic and diastolic) was significantly 42 higher in the third tertile of the dietary quality index than in the first tertile (OR = 1.42). The odds of a high fasting blood glucose level (mg/dl) was significantly 99% higher in the third tertile than in the first tertile (OR = 1.99; 95% CI: 1.58–2.52). In addition, the odds of high SGPT in the third tertile compared to the first tertile increased significantly by 52% (OR = 1.52; 95% CI: 1.11–2.06).

**Table 4:** The association between metabolic risk factors and DQI-I tertile using binary logistic regression

Variables	Tertile 1	Tertile 2	Tertile 3	*P*-trend
	Odds ratio (95% CI)			
**Body mass index** (kg/m^2^)	1	**1.163 (1.030–1.311)**	**1.443 (1.271–1.367)**	**< 0.001**
Model 1	1	**1.157 (1.205–1.305)**	**1.434 (1.263–1.627)**	**< 0.001**
Model 2	1	1.122 (0.991–1.271)	**1.332 (1.157–1.511)**	**< 0.001**
**Waist circumference** (cm)	1	0.931 (0.831–1.041)	**0.854 (0.761–0.958)**	**0.027**
Model 1	1	0.948 (0.811–1.106)	**0.729 (0.621–0.855)**	**< 0.001**
Model 2	1	1.090 (0.928–1.280)	0.944 (0.798–1.280)	**< 0.001**
**Waist-to-hip ratio**	1	**0.865 (0.772–0.966)**	**0.807 (0.719–0.904)**	**< 0.001**
Model 1	1	0.870 (0.754–1.003)	**0.738 (0.636–0.854)**	**< 0.001**
Model 2	1	0.983 (0.848–1.139)	0.936 (0.802–1.093)	**< 0.001**
**Waist-to-height ratio**	1	0.968 (0.786–1.190)	**1.256 (1.002–1.572)**	0.055
Model 1	1	**0.739 (0.563–0.970)**	0.904 (0.670–1.220)	**< 0.001**
Model 2	1	0.783 (0.549–1.033)	0.992 (0.728–1.351)	**< 0.001**
**Body fat percentage** (kg)	1	**0.460 (0.408–0.517)**	**0.240 (0.209–0.274)**	**< 0.001**
Model 1	1	**0.343 (0.302–0.391)**	**0.161 (0.138–0.187)**	**< 0.001**
Model 2	1	**0.271 (0.189–0.060)**	**0.107 (0.076–0.135)**	**< 0.001**
**Total cholesterol** (mg/dl)	1	0.976 (0.825–1.121)	0.871 (0.746–1.015)	0.198
Model 1	1	0.940 (0.802–1.101)	**0.840 (0.715–0.987)**	**< 0.001**
Model 2	1	0.951(0.811–1.115)	0.858 (0.727–1.012)	**< 0.001**
**Triglyceride** (mg/dl)	1	1.700 (0.998–1.369)	**1.224 (1.042–1.435)**	**0.031**
Model 1	1	1.105 (0.940–1.299)	1.117 (0.947–1.319)	**< 0.001**
Model 2	1	1.089 (0.925–1.282)	1.086 (0.917–1.286)	**< 0.001**
**HDL- cholesterol** (mg/dl)	1	**1.206 (1.780–1.349)**	**1.194 (1.050–1.339)**	**0.001**
Model 1	1	**1.196 (1.063–1.343)**	1.150 (0.019–1.296)	**< 0.001**
Model 2	1	**1.140 (1.112–1.638)**	1.193 (0.930–1.391)	**< 0.001**
**LDL – cholesterol** (mg/dl)	1	0.042 (0.691–1.566)	0.873 (0.691–1.350)	0.718
Model 1	1	1.075 (0.704–1.637)	0.957 (0.610–1.500)	**< 0.001**
Model 2	1	1.128 (0.738–1.725)	1.055 (0.666–1.672)	**< 0.001**
**Systolic blood pressure** (mmHg)	1	1.127 (0.965–1.314)	**1.313 (1.126–1.531)**	**0.002**
Model 1	1	0.999 (0.992–1.386)	**1.392 (1.175–1.646)**	**< 0.001**
Model 2	1	1.190 (0.006–1.407)	**1.429 (1.203–1.698)**	**< 0.001**
**Diastolic blood pressure** (mmHg)	1	1.152 (0.943–1.404)	**1.407 (1.157–1.709)**	**0.002**
Model 1	1	1.125 (0.913–1.383)	**1.372 (1.117–1.683)**	**< 0.001**
Model 2	1	1.145 (0.930–1.411)	**1.426 (1.157–1.758)**	**< 0.001**
**Fast blood sugar** (mg/dl)	1	1.041 (0.815–1.327)	**1.885 (1.511–2.350)**	**< 0.001**
Model 1	1	1.084 (0.822–1.390)	**1.999 (1.585–2.520)**	**< 0.001**
Model 2	1	0.927 (0.617–1.392)	1.275 (0.868–1.870)	**< 0.001**
**Aspartate aminotransferase** (IU/L)	1	1.024 (0.758–1.383)	1.096 (0.810–1.482)	0.828
Model 1	1	1.034 (0.754–1.393)	1.011 (0.784–1.462)	**0.019**
Model 2	1	1.030 (0.756–1.402)	1.078 (0.078–1.482)	0.817
**Alanine aminotransferase** (IU/L)	1	**1.433 (1.054–1.949)**	**1.520 (1.116–2.069)**	**0.014**
Model 1	1	1.288 (0.941–1.763)	1.322 (0.962–1.812)	**< 0.001**
Model 2	1	1.226 (0.895–1.681)	1.199 (0.868–1.656)	**< 0.001**
**Alkaline phosphatase** (IU/L)	1	0.685 (0.436–1.073)	0.960 (0.631–1.457)	0.205
Model 1	1	0.703 (0.442–1.117)	1.032 (0.669–1.591)	**< 0.001**
Model 2	1	0.753 (0.472–1.202)	1.189 (0.762–1.856)	**0.004**
**Gamma glutamyl transferase** (IU/L)	1	0.853 (0.660–1.099)	0.140 (0.887–1.441)	0.100
Model 1	1	0.850 (0.654–1.103)	1.164 (0.906–1.494)	**0.001**
Model 2	1	0.887 (0.682–1.154)	1.262 (0.976–1.631)	**0.002**

Model 1: Adjusted for age, sex, BMI, and energy intake

Model 2: Adjusted for age, sex, BMI, energy intake, socioeconomic status, and physical activity

### Distribution of metabolic risk factors by subgroups of DQI-I score

The variety subgroup was associated with BMI, WC, WHR, BFP, TC and FBS. Thus, the mean veriaty subgroup score was significantly higher in overweight/obese individuals, individuals with high FBS, and individuals with high FBS. The adequacy subgroup was significantly associated with BMI, WC, WHR, BFP, TG, HDL-C, FBS and SGPT. Accordingly, the adequacy score was significantly higher in overweight/obese individuals, individuals with high BFP, high TC, high FBS and high SGPT. The association between the moderation subgroup and risk factors was highly variable, and WC, WHR, WHtR, BFP, HDL-C, SBP, DBP, and FBS were associated with moderation. Thus, the moderation score was significantly higher only for FBS in the healthy range. In the overall balance subgroup, there was a significant association between anthropometric indicators such as WC, WHR and BFP as well as FBS and SGPT. The overall balance score was significantly higher for WC, WHR, BFP and FBS in the healthy range. In addition, there was a significant association between the DQI-I total score and anthropometric indicators, TC, TG, HDL-C, SBP, DBP, FBS and SGPT. Thus, the DQI-I total score was significantly higher for WC, WHR, BFP and TC in the healthy range (Table 5).

**Table 5:** The distribution of metabolic risk factors by subgroups of DQI-I score

Variables	Definition	*N*	Variety	Adequacy	Moderation	Overall balance	Total DQI-I score
**Body mass index** (kg/m^2^)	< 25	2124	12.24 ± 4.93	32.80 ± 3.99	11.76 ± 5.52	2.92 ± 2.24	59.24 ± 8.75
	> 25	4915	12.85 ± 4.68	33.50 ± 3.52	11.99 ± 5.54	2.82 ± 2.18	61.18 ± 8.37
P.value			**< 0.001**	**< 0.001**	0.115	0.080	**< 0.001**
**Waist circumference** (cm)	< 95	3124	13.04 ± 4.80	33.42 ± 3.76	11.60 ± 5.51	2.96 ± 2.19	61.04 ± 8.52
	> 95	3985	12.38 ± 4.72	33.18 ± 3.62	12.15 ± 5.54	2.77 ± 2.21	60.51 ± 8.49
P.value			**< 0.001**	**0.005**	**< 0.001**	**< 0.001**	**0.008**
**Waist-to-hip ratio**	< 0.95	3646	13.03 ± 4.79	33.42 ± 3.65	11.74 ± 5.57	2.94 ± 2.19	61.14 ± 8.44
	> 0.95	3393	12.27 ± 4.71	33.16 ± 3.71	12.2 ± 5.49	2.76 ± 2.20	60.32 ± 8.56
P.value			**< 0.001**	**0.003**	**0.004**	**0.001**	**< 0.001**
**Waist-to-height ratio**	< 0.5	525	13.03 ± 4.90	33.24 ± 3.76	11.02 ± 5.39	3.02 ± 2.21	60.32 ± 8.41
	> 0.5	6512	12.64 ± 4.76	33.30 ± 3.68	12.00 ± 5.54	2.84 ± 2.20	60.78 ± 8.51
P.value			0.071	0.742	**< 0.001**	0.077	0.227
**Body fat percentage** (kg)	< 29.5	4883	12.07 ± 4.73	33.15 ± 3.70	13.71 ± 4.95	3.41 ± 2.18	62.36 ± 8.06
	> 29.5	2232	13.99 ± 4.58	33.57 ± 3.64	7.98 ± 4.64	1.64 ± 1.71	57.20 ± 8.39
P.value			**< 0.001**	**< 0.001**	**< 0.001**	**< 0.001**	**< 0.001**
**Total cholesterol** (mg/dl)	< 150	1154	12.92 ± 4.82	33.43 ± 3.79	12.00 ± 5.60	2.86 ± 2.15	61.23 ± 8.59
	> 150	5915	12.62 ± 4.75	33.25 ± 3.66	11.90 ± 5.52	2.86 ± 2.22	60.64 ± 8.50
P.value			**0.045**	0.133	0.568	0.990	**0.032**
**Triglyceride** (mg/dl)	< 200	5997	12.62 ± 4.78	33.24 ± 3.71	11.89 ± 5.54	2.85 ± 2021	60.62 ± 8.58
	> 200	1072	12.90 ± 4.64	33.51 ± 3.53	12.05 ± 5.53	2.90 ± 2.17	61.37 ± 8.14
P.value			0.084	**0.030**	0.392	0.510	**0.008**
**HDL- cholesterol** (mg/dl)	> 45	3529	12.58 ± 4.81	33.13 ± 3.74	11.77 ± 5.58	2.84 ± 2.21	60.33 ± 8.68
	< 45	3539	12.75 ± 4.71	33.43 ± 3.62	12.06 ± 5.49	2.88 ± 2.20	61.14 ± 8.32
P.value			0.114	**< 0.001**	**0.030**	0.433	**< 0.001**
**LDL- cholesterol** (mg/dl)	< 160	6939	12.68 ± 4.76	33.29 ± 3.68	11.91 ± 5.54	2.85 ± 2.20	60.74 ± 8.52
	> 160	129	11.65 ± 4.74	32.70 ± 4.06	12.69 ± 4.98	3.13 ± 2.31	60.48 ± 8.26
P.value			0.085	0.070	0.109	0.162	0.732
**Systolic blood pressure** (mmHg)	< 12	5959	12.68 ± 4.79	33.23 ± 3.69	11.72 ± 5.47	2.85 ± 2.19	60.58 ± 8.51
	> 12	1156	12.65 ± 7.66	33.11 ± 3.66	12.89 ± 5.75	2.90 ± 2.24	61.57 ± 8.47
P.value			0.865	0.087	**< 0.001**	0.451	**< 0.001**
**Diastolic blood pressure** (mmHg)	< 8	6454	12.65 ± 4.78	33.29 ± 3.68	11.83 ± 5.51	2.85 ± 2.20	60.64 ± 8.51
	> 8	661	12.88 ± 4.64	33.23 ± 3.77	12.72 ± 5.67	2.90 ± 2.20	61.74 ± 8.41
P.value			0.236	0.688	**< 0.001**	0.611	**0.001**
**Fast blood sugar** (mg/dl)	< 125	6585	12.61 ± 4.78	33.25 ± 3.70	11.82 ± 5.48	2.87 ± 2.21	60.57 ± 8.50
	> 125	484	13.45 ± 4.48	33.73 ± 3.43	13.30 ± 6.02	2.64 ± 2.17	63.14 ± 8.57
P.value			**< 0.001**	**0.005**	**< 0.001**	**0.024**	**0.001**
**Aspartate aminotransferase** (IU/L)	> 9.5, < 39.5	6809	12.67 ± 4.77	33.28 ± 3.68	11.91 ± 5.53	2.85 ± 2.20	60.73 ± 8.52
	< 9.5, > 39.5	260	12.54 ± 4.61	33.38 ± 3.68	12.10 ± 5.57	2.95 ± 2022	60.99 ± 8.36
P.value			0.668	0.651	0.591	0.495	0.629
**Alanine aminotransferase** (IU/L)	> 7, < 55	6804	12.64 ± 4.77	33.26 ± 3.68	11.92 ± 5.55	2.85 ± 2.21	60.68 ± 8.54
	< 7, > 55	264	13.21 ± 4.46	33.84 ± 3.77	12 ± 5.13	3.17 ± 2.06	62.23 ± 7.73
P.value			0.056	**0.012**	0.819	**0.019**	**0.003**
**Akaline phosphatase** (IU/L)	< 350	6947	12.67 ± 4.76	33.28 ± 3.69	11.91 ± 5.54	2.85 ± 2.21	60.37 ± 8.52
	> 350	122	12.37 ± 4.74	33.15 ± 3.54	12.41 ± 5.44	3.01 ± 2.17	60.968.27
P.value			0.492	0.691	0.319	0.436	0.768
**Gamma glutamyl transferase** (IU/L)	< 63.5	6680	12.66 ± 4.77	33.27 ± 3.69	11.90 ± 5.55	2.86 ± 2.21	60.70 ± 8.52
	> 63.5	388	12.83 ± 4.60	33.42 ± 3.56	12.20 ± 5.28	2.85 ± 2.19	61.31 ± 8.38
P.value			0.485	0.464	0.295	0.950	0.170

The values of the variables were reported as the mean and standard deviation of the scores of the subgroups of the DQI-I. The p-values are obtained through a t-test

### Association between metabolic risk factors and subgroups of DQI-I score

In the variety subgroup, an increase of one unit resulted on average in a decrease in BFP, TC, HDL-C, LDL-C (*P* < 0.001) and an increase in BMI, WC, TG, FBS, SGPT and GGT (*P* < 0.05). Furthermore, in the adequacy subgroup, a one-unit increase in the subgroup scores, TC, HDL-C and LDL-C led to their decrease (β=-0.49 mg/dl, β=-0.31 mg/dl and β=-0.35 mg/dl, respectively; *P* < 0.001). Conversely, BMI, WC, BFP, TG, FBS, SGPT and GGT increased significantly (*P* < 0.05). When the score in the moderation subgroup increased by one unit, the WC, BFP, TG. LDL-C, SBP, DBP and FBS increased by 0.07 cm, 0.12%, 0.40 mg/dl, 0.15 mg/dl, 0.27 mmHg, 0.11 mmHg and 0.06 mg/dl, which are significant. When looking at the overall balance subgroup, an increase of one unit led to a regular decrease in BFP (β=-0.17%) and HDL-C (β=-0.16 mg/dl). In addition, SBP and DBP showed a significant positive association in the moderation subgroup (β = 0.28 and β = 0.12 mg/dl). A one-unit increase in total DQI-I led to a decrease in BFP (β=-0.03%), TC (β=-0.19 mg/dl), HDL-C (β=-0.15 mg/dl) and LDL-C (β=-0.96 mg/dl). Conversely, BMI, WC, TG, SBP, DBP, FBS, SGPT and GGT increased significantly (*P* < 0.05) (Table 6).

**Table 6:** The association between metabolic risk factors and subgroups of DQI-I score using linear regression

Variables	Variety			Adequacy			Moderation			Overall balance			Total DQI-I score		
	β	S.E	*P*	β	S.E	*P*	β	S.E	*P*	β	S.E	*P*	β	S.E	*P*
BMI	**0.047**	**0.011**	**< 0.001**	**0.109**	**0.015**	**< 0.001**	0.017	0.10	0.087	-0.048	0.025	0.055	**0.039**	**0.006**	**< 0.001**
WC	**0.070**	**0.026**	**0.007**	**0.181**	**0.034**	**< 0.001**	**0.079**	**0.022**	**< 0.001**	-0.069	0.056	0.222	**0.085**	**0.014**	**< 0.001**
WHR	**0.000**	**0.000**	**< 0.001**	**0.0001**	**0.000**	**< 0.001**	0.000	0.000	0.246	6.06e	0.000	0.985	**0.000**	**0.000**	**< 0.001**
WHtR	**-0.000**	**0.000**	**< 0.001**	-0.000	0.000	0.117	**0.000**	**0.000**	**< 0.001**	**-0.001**	**0.000**	**0.004**	-0.000	0.000	0.643
BFP	**-0.177**	**0.023**	**< 0.001**	**0.089**	**0.030**	**0.003**	**0.122**	**0.020**	**< 0.001**	**-0.170**	**0.051**	**< 0.001**	**-0.032**	**0.013**	**0.015**
TC	**-0.548**	**0.094**	**< 0.001**	**-0.494**	**0.122**	**< 0.001**	0.156	0.081	0.056	0.123	0.205	0.547	**-0.190**	**0.531**	**< 0.001**
TG	**0.532**	**0.204**	**0.009**	**1.070**	**0.263**	**< 0.001**	0.408	0.175	**0.020**	0.278	0.440	0.527	**0.558**	**0.114**	**< 0.001**
HDL-C	**-0.257**	**0.028**	**< 0.001**	**-0.317**	**0.036**	**< 0.001**	-0.16	0.024	0.509	**-0.164**	**0.060**	**0.007**	**-0.158**	**0.015**	**< 0.001**
LDL-C	**-0.338**	**0.063**	**< 0.001**	**-0.356**	**0.081**	**< 0.001**	**0.152**	**0.054**	**0.005**	0.177	0.126	0.195	**-0.960**	**0.35**	**0.046**
SBP	-0.042	0.041	0.308	-0.060	0.053	0.262	**0.279**	**0.035**	**< 0.001**	**0.285**	**0.090**	**0.001**	**0.112**	**0.023**	**< 0.001**
DBP	-0.014	0.024	0.542	0.036	0.031	0.242	**0.112**	**0.20**	**< 0.001**	**0.126**	**0.052**	**0.009**	**0.044**	**0.013**	**0.001**
FBS	**0.254**	**0.075**	**0.001**	**0.203**	**0.097**	**0.036**	**0.320**	**0.064**	**< 0.001**	-0.299	0.162	0.066	**0.233**	**0.042**	**< 0.001**
SGOT	0.036	0.022	0.105	0.024	0.029	0.412	-0.025	0.019	0.191	0.062	0.049	0.208	0.009	0.012	0.461
SGPT	**0.327**	**0.035**	**< 0.001**	**0.398**	**0.046**	**< 0.001**	-0.036	0.030	0.235	0.109	0.077	0.157	**0.169**	**0.020**	**< 0.001**
ALP	-0.089	0.151	0.556	-0.085	0.196	0.661	0.145	0.130	0.265	0.376	0.327	0.250	0.043	0.084	0.610
GGT	**0.246**	**0.045**	**< 0.001**	**0.246**	**0.058**	**< 0.001**	-0.014	0.039	0.718	0.156	0.097	0.110	**0.128**	**0.025**	**< 0.001**

Regression coefficient (β), standard error of coefficient (S.E), and p-value are presented using linear regression model

BMI: Body mass index (kg/m^2^)/ WC: Waist circumference (cm)/ WHR: Waist-to-hip ratio / WHtR: Waist-to-height ratio / BFP: Body fat percentage (kg)/ TC: Total cholesterol (mg/dl)/ TG: Triglyceride (mg/dl)/ HDL-C: High density lipoprotein - cholesterol (mg/dl)/ LDL-C: Low density lipoprotein - cholesterol (mg/dl)/ SBP: Systolic blood pressure (mmHg)/ DBP: Diastolic blood pressure (mmHg)/ FBS: Fast blood sugar (mg/dl)/ SGOT: Aspartate aminotransferase (IU/L)/ SGPT: Alanine aminotransferase (IU/L)/ ALP: Alkaline phosphatase (IU/L)/ GGT: Gamma glutamyl transferase (IU/L)

## Discussion

This study showed that participants’ DQI-I scores were generally low, with more than one-third scoring the lowest level of DQI-I. Furthermore, the association between DQI-I and metabolic factors was complex. However, examination of the total DQI-I score and its subgroups revealed that DQI-I and the variety and adequacy subgroups had a significant negative association with TC. A one-unit increase in DQI-I total score resulted in a 0.19 decrease in TC. DQI-I and its subgroups also showed a significant positive association with BMI.

In this study, there was an inverse association between DQI-I scores and TC. A review by Alkervi et al. showed an inverse relationship between DQI-I score and TC [15]. No association between TC and DQI-I was observed in many studies [19–21]. The likely explanation for this inverse relationship is that higher DQI-I scores are associated with lower total fat and cholesterol intake. Studies have shown that a reduction in fat and cholesterol consumption leads to a decrease in TC levels [15, 22].

Our study showed a significant positive association between FBS and total DQI-I and its subgroups. However, it also revealed that the FBS level was lower in the second tertile compared to the other tertiles. A similar result was reported by Karimi et al. [23]. This is due to higher consumption of fiber and antioxidants in the second tertile and higher consumption of energy and refined grains in the third tertile [23]. Studies found that higher DQI-I score was linked to lower blood glucose [12, 15]. Fruit and vegetable consumption is strongly associated with a lower risk of type 2 diabetes [24]. However, the differences in the study results could be due to the fact that people with high DQI-I scores consume more energy-rich foods. In addition, only the FBS score was used to assess blood glucose in this study, whereas the DQI-I score may also be related to other blood glucose indicators.

In this study, a positive association was found between DQI-I values and SBP and DBP. Such a association has also been found in other studies [20, 25], with the exception that one study found higher blood pressure in men with lower DQI-I adherence [26]. The DQI-I score also showed a negative association with HDL-C. While some studies found decreasing HDL-C with higher DQI-I score [12, 15], one study found the opposite trend [27]. The differences in results may be due to differences in sample size, methods and health status of participants.

There was a significant negative association between the DQI-I variety subgroup and TC. The survey by Mahdavi Roshan et al. showed a positive association between dietary variety and some CVD risk factors [28]. A meta-analysis showed that there was no association between dietary variety index and TC [29]; however, one study found a moderate correlation between dietary variety and the DQI-I variety index subgroup [30]. Eating a wide range of food groups is associated with a more favorable TC [31]. A varied diet increases levels of key micronutrients such as vitamin C, calcium and fiber. Fiber from vegetables, fruits and whole grains helps to reduce cholesterol absorption and thus lower total cholesterol levels [32, 33].

In the adequacy subgroup, there was a significant inverse association between LDL-C and TC. The key reason is dietary fiber, which lowers LDL-C [34]; Fruits and vegetables, rich in fiber, reduce both TC and LDL-C [35]. People with normal blood lipids consume more vegetables and beans than people with dyslipidemia [31]. Fiber can be fermented in the colon by microflora and produce short-chain fatty acids (SCFAs), which help reduce CVD risk factors by inhibiting cholesterol synthesis [36, 37]. In addition, the consumption of fruit and vegetables increases the antioxidant capacity of the serum and thus protects against lipid peroxidation [38]. Protein type also influences blood lipids: animal protein increases TC and LDL-C, while vegetable protein lowers the LDL-C to HDL-C ratio [39].

The DQI-I score has a significant inverse association with the WC, WHR, and BFP and a significant positive association with BMI. Studies showed that better diet quality leads to lower obesity [40, 41]. Some studies showed that increased dietary quality was associated with higher obesity in adults [42, 43]. A high-quality diet is rich in fiber, vitamins, and antioxidants while reducing fats, sodium, and sugars, contributing to anti-obesity effects [44]. Diets high in energy and fat impair appetite control and are associated with obesity [33, 45]. The Western diet, which is high in saturated fat and low in fruit and fiber, contributes to inflammation and higher levels of body fat. The mechanism linking saturated fat intake to increased BFP is unclear, but pro-inflammatory cytokines such as IL-1, IL-6 and TNFα may increase appetite and caloric intake in obese individuals [46]. In this study, the direct association between BMI and DQI-I may reflect weight gain, possibly due to muscle mass, as DQI-I is inversely related to BFP. In addition, higher DQI-I scores are associated with lower WC and WHR, suggesting lower central obesity.

The variety subgroup of DQI-I had a negative and significant association with WC and WHR. Azadbakht et al.‘s study confirmed the findings of this study by showing the inverse association between variety and abdominal obesity [47]; Abris et al. also reported an inverse association between variety with WC and BMI [48]. In a systematic review study, no association was observed between variety and body fat, and the cause was reported to be the positive association between variety and increased calorie intake [49]. However, the higher energy intake associated with a higher DQI-I score comes mainly from low-energy foods such as fruits, vegetables and whole grains [50]. Greater dietary variety also leads to lower consumption of fast food, which is associated with abdominal obesity [47]. More variety increases protein and antioxidant intake, maintains muscle and reduces body fat, leading to lower general and abdominal obesity [51].

An inverse and significant association was observed between the adequacy subgroup with WHR. Studies have shown that fiber intake is associated with a reduction in visceral adipose tissue [52, 53]. These foods promote slower food intake, faster satiety, reduced energy food intake, and better digestion through increased bile acid secretion and gastrointestinal motility, which improves body fat distribution and weight [54]. It showed that dietary calcium is inversely related to the occurrence of obesity in people [52]. Calcium binds to fatty acids, preventing fat absorption and reducing calorie intake. It also stimulates fat breakdown and reduces fat formation [54].

In the overall balance subgroup, there was a significant association with BFP. During a 9-year follow-up, Lofley et al. showed that the quality and quantity of macronutrients were related to changes in Anthropometric indices [55]. A higher carbohydrate intake is associated with a lower body fat mass, while increased fat consumption is associated with a reduction in muscle mass due to hormonal changes; higher fat intake can reduce testosterone, estrogen and growth hormone [56]. Optimizing macronutrient composition is critical for body composition management. Consumption of MUFA and PUFA reduces fat deposition compared to SFA by promoting β-oxidation of fatty acids, improving TEE and reducing BMI [57].

This study examined the association between metabolic risk factors and DQI-I. The strengths are the use of data from the RaNCD, which contained complete information on the participants. Consequently, most confounding variables were controlled, resulting in high reliability of the reported results. However, as this was a cross-sectional study, it was not possible to examine cause-and-effect relationships. In addition, diet can change over time and these changes are difficult to measure. Moreover, this study focused on metabolic patients (cardiovascular patients and diabetics), and the incidence of metabolic patients remains unknown.

## Conclusion

This study highlights the concerning dietary quality of the participants, a significant proportion of whom fall into the lowest tertile of the DQI-I. The results showed that higher dietary quality is associated with better metabolic outcomes, but paradoxically also with an increased risk of overweight and obesity. Remarkably, men had better dietary quality than women, indicating a potential area for targeted intervention. The results suggest that better diet quality may help to control TC and BFP levels. Although a better diet cannot optimize all metabolic risk factors, it is crucial for overall health. Future large-scale prospective cohort or clinical studies should investigate the specific dietary components that influence these associations and the underlying mechanisms. To optimize health outcomes, it is crucial to implement targeted interventions that address both dietary quality and lifestyle factors, especially for populations at risk. Public health initiatives should aim to promote balanced diets rich in nutrients while considering gender differences in dietary habits to foster overall health improvements.

## Data Availability

The data analyzed in the study are available from the corresponding author upon reasonable request.

## Abbreviations

ALP: Alkaline Phosphatase
BFP: Body Fat Percentage
BMI: Body Mass Index
CDC: Centers for Disease Control and Prevention
DBP: Diastolic Blood Pressure
DQI: I-Dietary Quality Index-International
FBS: Fast Blood Sugar
FFQ: Food Frequency Questionnaire
FVS: Food Variety Score
GGT: Gamma Glutamyl Transferase
HDL: High-Density Lipoprotein
LDL: Low-Density Lipoprotein
MUFA: Monounsaturated Fatty Acids
NCDs: Non-Communicable Diseases
PUFA: Polyunsaturated Fatty Acids
RaNCD: Ravansar Non-Communicable Diseases
SBP: Systolic Blood Pressure
SES: Socio-Economic Status
SGOT: Aspartate Aminotransferase
SGPT: Alanine Aminotransferase
TC: Total Cholesterol
TG: Triglyceride
T2D: Type 2 Diabetes
WC: Waist Circumference
WHR: Waist to Hip Ratio
WHtR: Waist to Height Ratio
